# Influence of Ultrasound-Assisted Vacuum Drying on Physicochemical Characteristics, Antioxidant Activity, and α-Glucosidase Inhibition Activity of *Flos Sophorae Immaturus*

**DOI:** 10.3390/foods12030671

**Published:** 2023-02-03

**Authors:** Yuhong Gong, Jun Li, Jinwei Li, Liuping Fan, Li Wang

**Affiliations:** 1 State Key laboratory of Food Science & Technology, Jiangnan University, 1800 Lihu Avenue, Wuxi 214122, China; 2 School of Food Science and Technology, Jiangnan University, 1800 Lihu Avenue, Wuxi 214122, China; 3 Collaborat Innovat Ctr Food Safety & Qual Control, Jiangnan University, 1800 Lihu Avenue, Wuxi 214122, China

**Keywords:** ultrasonic-assisted vacuum drying, *Flos Sophorae Immaturus*, flavonoids, antioxidant activity, α-glucosidase inhibition activity

## Abstract

*Flos Sophorae Immaturus* (FSI) contains a large number of bioactive substances with antioxidant and hypoglycaemic activity. However, a feasible drying process plays an important role in the retention of its biological activity. The present work investigated the effects of ultrasound-assisted vacuum drying (UAVD) on FSI samples in terms of drying time, colour, microstructure, and total flavonoid content (TFC). Meanwhile, the antioxidant activity and α-glucosidase inhibition activity were also evaluated. The results show that the drying time of UVAD samples was decreased by 40% compared to that of the single vacuum-dried (VD) samples (600 W for 10 min). The cellular porous structures of FSI tissue were formed by UAVD, which promoted the migration of water from the inside to the outside. Furthermore, samples treated by UAVD exhibited better antioxidant activities and α-glucosidase and α-amylase inhibition capacities, with DPPH (81.86%), ABTS (88.61%), FRAP (83.05%), α-glucosidase inhibition capacity (89%), α-amylase (85%), drying time (3 h), and total aberration (Δ*E*) (1.63) being the highest characteristic traits. In this condition, the highest levels of total flavonoid content (TFC), rutin, quercetin, kaempferol, isorhamnetin, and genistein were obtained with 266.94, 239.46, 35.56, 8.54, 10.37, and 5.64 mg/g DW, respectively. The results confirm that UAVD is a novel method that significantly reduced the VD time and promoted the release of the bioactive substances of FSI.

## 1. Introduction

*Flos Sophorae Immaturus* (FSI) is a traditional Chinese food [1]. FSI contains a large number of flavonoids (e.g., rutin, quercetin, kaempferol, etc.) [2], small amounts of phenolic acids (e.g., protocatechuic acid, chlorogenic acid, carotenoids, etc.), and a few other ingredients (e.g., carotenoids, curcumin, anthocyanin, chlorophyll, etc.) [1]. With a noteworthy rutin content of up to 20% [3], FSI has exhibited a range of health benefits such as hypoglycaemic, antioxidant, anti-ageing, anti-allergic, and anti-cancer properties [4]. Therefore, FSI is a promising raw material for phytochemicals and has great potential for further development as a raw material for health products [5]. However, the quantity and quality of these bioactive substances can be negatively impacted during postharvest drying [6]. Therefore, it is of significance to use appropriate drying methods and rational handling to enhance the extraction of these bioactive substances.

Vacuum drying (VD) is widely used for the drying of various plant materials such as fruits and vegetables [7]. VD is suitable for substances with low vacuum and easy reaction with oxygen, and it has the advantages of low temperature and absence of oxygen and has a high preservation rate of biologically active ingredients [8]. However, VD requires longer drying times and higher energy consumption due to the lack of air and the difficulty of heat convection [6]. With the advantages of high efficiency, low cost, environmental friendliness, and flexibility in combination with other treatment processes, ultrasound technology has widespread application prospects in various industrial applications such as drying and extraction [9]. Ultrasound can enhance the internal moisture transfer process and create cavitation, which facilitates the removal of strongly adhering moisture without significantly heating the product so that heat-sensitive food ingredients can be preserved [10]. At present, ultrasound has been applied to the drying of fruits and vegetables and has shown good results in terms of drying speed and product quality [11,12,13]. However, it is still unknown whether the application of UAVD possesses these benefits.

Therefore, the present study aimed to explore the effects of ultrasound-assisted vacuum-dried (UAVD) FSI by analysing the microstructure, colour, drying rate, flavonoids, antioxidant activity, α-glucosidase, and α-amylase inhibitory activities. Our findings provide insight into the advantages of applying UAVD as opposed to VD, offering a promising drying method for FSI applications.

## 2. Materials and Methods

### 2.1. Materials and Reagents

FSI was purchased from Hebei Anguo Yao Yuan Trading Co., Ltd. (Baoding, China). 1,1-Diphenyl-2-picrylhydrazyl (DPPH), 2,2′-Azino-bis (3-ethylbenzothiazoline-6-sulphonic acid) (ABTS), Tri-2-pyridyl-s-triazine (TPTZ), rutin, quercetin, kaempferol, isorhamnetin, genistein, and ethanol were obtained from the China National Pharmaceutical Foreign Trade Corporation (Shanghai, China). The α-glucosidase (50 U/mg) and α-amylase (50 U/mg) were purchased from Yuanye Biological Technology Co., Ltd. (Shanghai, China). All chemicals used in this study were of analytical grade.

### 2.2. Ultrasound Treatments and VD Methods

Fresh FSI was performed using an ultrasonic reactor chamber (TL615HTD, Jiangsu Tenlin Instrument Co., Ltd., Yancheng, China). The experimental design included two factors, ultrasound power and ultrasound time, with four levels of ultrasound power (150, 300, 450, and 600 W) and three levels of ultrasound time (5, 10, and 15 min), for a total of 12 experimental treatments. The ultrasonic medium was distilled water.

The FSI after ultrasonic treatment was performed using a VD oven (DP33C, Yamato, Tokyo, Japan). The FSI sample was placed on the wire netting of the drying oven, the vacuum degree was maintained at −0.098 mbar, and the temperature was maintained at 60 °C for 5 h. The final moisture content of the FSI sample was 11%. All experimental treatments were performed in triplicate.

### 2.3. Moisture Content (MC)

The moisture content was determined according to the method of Gong et al. [4] with minor alterations. About 3 g of FSI samples was placed in an open aluminium box (15 mL) and dried in a hot-air drying oven at 105 °C until the constant weight was achieved. The data were expressed as g/100g wet basis.

### 2.4. Stereomicroscope (SM)

The visual characterization was conducted using stereo microscopy (M205C, Leica, Ltd., Frankfurt, Germany) according to the procedure of Liu et al. [14] with minor alterations. Briefly, 1 mm off the top of the FSI was cut. The section morphology and surface morphology of FSI were observed at 2× objective and 5× objective.

### 2.5. Scanning Electron Microscopy (SEM)

The microstructure was analysed using the SEM instrument (S-4800, Hitachi, Ltd., Tokyo, Japan) with a magnification of 300× and 1000× according to Liu et al. [15]. The FSI samples should be dried first, and then placed in a gold sprayer to be coated with gold palladium for the SEM observation.

### 2.6. Colour

The colour was determined by a spectrophotometer (CM-2300D, Konica Minolta, Tokyo, Japan) according to the method of Yao et al. [16] with minor alterations. This method is based on the CIE-L*a*b* system, where L* corresponds to the brightness value, a* is the green/red value, and b* is the blue/yellow value. The total aberration (Δ*E*) represents the total colour difference in the samples and is calculated using Equation (1).

(1) $$\Delta E=\sqrt{{\left(L^{*}-L_{0}^{*}\right)}^{2}+{\left(a^{*}-a_{0}^{*}\right)}^{2}+{\left(b^{*}-b_{0}^{*}\right)}^{2}}$$

where Δ*E* is the total aberrations; $L^{*}$ and $L_{0}^{*}$ are the luminance values of the samples and controls, respectively; $a^{*}$ and $a_{0}^{*}$ are the green/red values of the samples and controls, respectively; and $b^{*}$ and $b_{0}^{*}$ are the blue/yellow values for the samples and controls, respectively.

### 2.7. Total Flavonoid Contents (TFC)

The TFC was measured by UV−vis spectrophotometer according to the method of Yu et al. [6]. FSI extract (1.0 mL) was mixed with 0.3 mL of 5% NaNO_2_ (*w*/*w*) and incubated at 25 °C for 5 min. Then, 0.3 mL of 10% AlCl_3_ (*w*/*w*) was added to the mixture and incubated at 25 °C for 6 min. The mixture was added to 4 mL of 1 M NaOH and supplemented to 10 mL with distilled water, then measured at 510 nm after being incubated at 25 °C for 12 min. Results were expressed as micrograms per gram of dry weight of FSI powder.

### 2.8. HPLC Analysis of Flavonoid Constituents

HPLC (1260, Agilent Technologies, Santa Clara, CA, USA) was performed to measure the contents of single phenolics [6]. The elution conditions were as follows: solvent A, consisted of 10% (*v*/*v*) acetonitrile solution and 0.1% (*w*/*v*) acetic acid; solvent B, consisted of 90% (*v*/*v*) acetonitrile solution with 0.1% (*w*/*v*) acetic acid. The procedure was performed as follows: 0–20 min, 35% B and 20–29 min, linear gradient to 100% B. The wavelength was fixed at 360 nm; flow rate, 0.6 mL/min; and sample injection volume, 20 μL.

### 2.9. Antioxidant Capacity Determination

The method for determination of DPPH, ABTS, and FRAP was that of Xiao et al. [17] with minor revisions.

The FSI extract (1.0 mL) was mixed with 1 mL of 0.2 mM DPPH reagent (in methanol solution) before incubation in dark conditions at room temperature for 20 min. The absorbance of samples was determined at λ = 517 nm. Distilled water was used as a blank. Different concentrations of Trolox (10–100 μg/mL) were measured as a standard curve and DPPH activity was expressed as the Trolox equivalent concentration.

The ABTS stock solution was first prepared and diluted until the absorbance was around 0.7 at λ = 734 nm. Next, 150 μL of sample or standard solution was added to 2.85 mL of ABTS working solution. The mixture was shaken vigorously and incubated in the dark for 6 min before the absorbance was determined at λ = 734 nm. Ethyl alcohol was used as the blank. Different concentrations of Trolox (10–100 μg/mL) were measured as a standard curve and ABTS activity was expressed as the Trolox equivalent concentration.

The FRAP reagent was mixed with 300 mM sodium acetate buffer, 10 mM TPTZ, and 20 mM ferric chloride solution in 40 mM hydrochloric acid at a ratio of 10:1:1 (*v*/*v*/*v*), respectively. Sample solutions (100 μL) were mixed with 1.9 mL of FRAP reagent, and then incubated at room temperature for 30 min. The absorbance was determined at λ = 593 nm. Distilled water was used as the blank control. Vc was measured at different concentrations (10–100 μg/mL) to produce a standard curve. The FRAP activity was then expressed as the Vc equivalent concentration. All measurements were performed in triplicate.

### 2.10. α-Glucosidase and α-Amylase Inhibition Activity

The α-glucosidase and α-amylase inhibition activities were measured according to the method of Ismail et al. [18]. The initial reading (T_0_) was recorded at 405 nm. To initiate the reaction, 20 μL of the stock solution of the α-glucosidase enzyme (1 unit mL^−1^ in PBS) was added followed by incubation at 37 °C for 10 min. Then, the final reading (T_10_) was determined again at 405 nm. Blank PBS subjected to the described procedure above without sample extracts was used as a negative control. The percentage of inhibitory activity was calculated using Equation (2):

(2) $$\mathrm{Inhibitory}\;\mathrm{activity}\;\left(\%\right)=\left(1-\frac{A_{sample}\left(T_{10}-T_{0}\right)}{A_{-ve\;control}\left(T_{10}-T_{0}\right)}\right)\times 100$$

Briefly, 20 μL of phosphate buffer (100 mM, pH 7), 10 μL of α-amylase (2 units mL^−1^), and a 50 μL sample at varying concentrations (0.1–0.5 mg mL^−1^) were pre-incubated for 20 min at 37 °C. After that, 20 μL of the substrate (1% soluble starch) was added and further incubated for 30 min at 37 °C. Then, 100 μL of the DNS (3,5-Dinitrosalicylic acid) colour reagent was added to stop the reaction, and the samples were boiled for 10 min. The absorbance of α-amylase inhibitory activity was measured at 540 nm and was calculated by Equation (3):

(3) $$\mathrm{Inhibitory}\;\mathrm{activity}\;\left(\%\right)=\left(\frac{A_{sample}-A_{-ve\;control}}{A_{-ve\;control}}\right)\times 100$$

where *A_-ve control_* is the absorbance of the negative control.

### 2.11. Statistical Analysis

Duncan’s multivariate range test in SPSS software (Version 20.0, IBM, Chicago, IL, USA) was used for statistical analysis. The statistical significance of *p* < 0.05 was evaluated by one-way ANOVA. All determinations were repeated three times.

## 3. Results and Discussion

### 3.1. Effect of UAVD on MC of FSI

**Figure 1:**
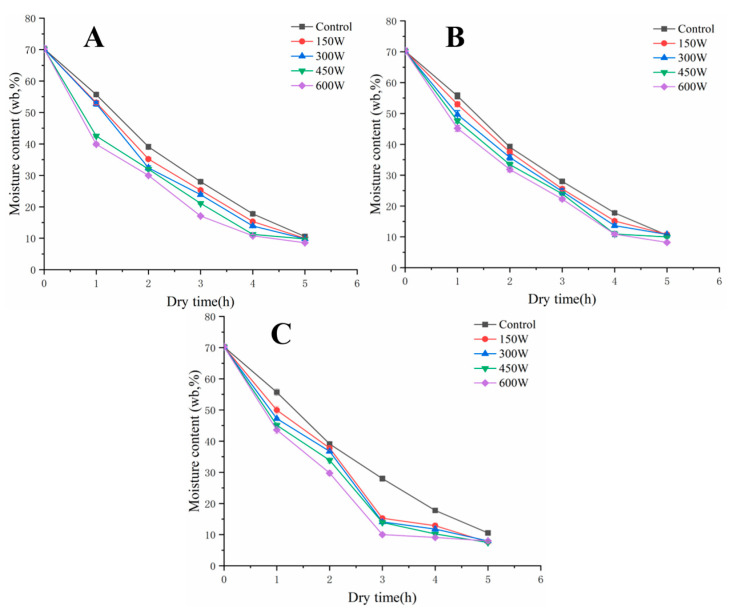
Changes in moisture content of FSI with ultrasonic time and power during VD. Ultrasonic time, 5 min (**A**); ultrasonic time, 10 min (**B**); ultrasonic time, 15 min (**C**). The control was soaked in FSI for the same time for VD. FSI, *Flos Sophorae Immaturus*. VD, vacuum drying.

The UAVD drying characteristics of FSI are described by the change in moisture ratio during the drying process [19]. The ultrasonic treatment increased the drying efficiency and accelerated the decreasing trend of MC [20,21]. The initial MC of the samples was 70.36% and the MC value gradually decreased with the increase in drying time. However, the MC values of the samples sonicated at 150, 300, 450, and 600 W for 5 min were lower than those of the VD samples (Figure 1A). This phenomenon was caused by the formation of microchannels after ultrasound treatment, which contributed to the rapid diffusion of water and prevented severe damage to the cellular structure [11]. Similar results were observed by Colucci et al. [8] during the ultrasound.

In Figure 1B, at an ultrasound time of 10 min, the VD time was reduced by 20% compared to the control sample after only a 450 W ultrasound was required. In contrast, at an ultrasound time of 15 min, drying was even faster. It is obvious that the drying time of samples treated by the UAVD at 600 W was shortened by 40% compared to that of the VD samples (Figure 1C). These results demonstrate that ultrasound time and power significantly affect the MC after the VD process. This is consistent with the study by Liu et al. [22], in which ultrasonic pre-treatment of *Platycodon grandiflorum* at 200 W ultrasonic power for 30 min caused shorter dehydration times compared to untreated samples.

### 3.2. Morphology Analysis

**Figure 2:**
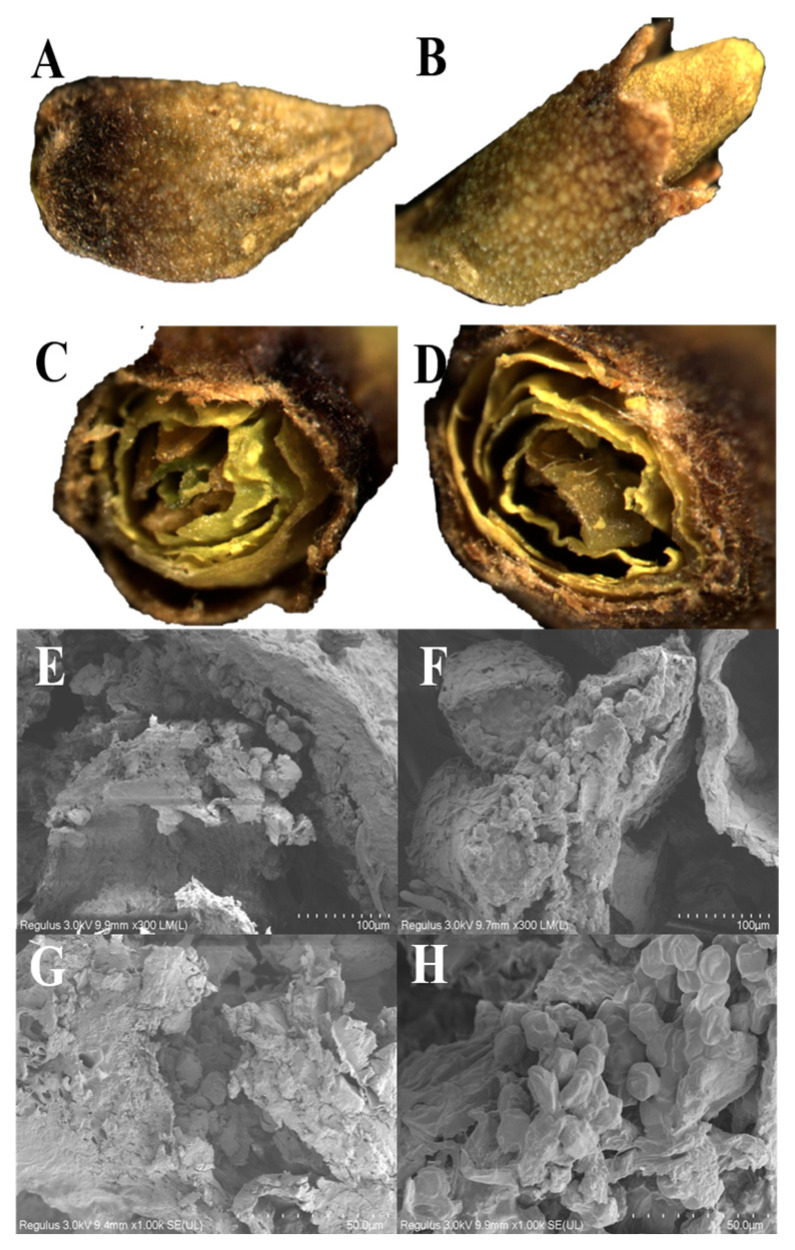
Stereomicroscope of (**A**) VD, 2×, (**B**) UAVD, 2×, (**C**) VD, 5×, and (**D**) UAVD, 5×. Scanning electron micrographs of (**E**) VD, 300×, (**F**) UAVD, 300×, (**G**) VD, 1000×, and (**H**) UAVD, 1000× at two different scales (0.1 mm and 50 μm), showing the structural characterization of the FSI after ultrasonic-assisted treatment. UAVD, ultrasonic-assisted vacuum drying.

The morphology of the FSI granules was observed by SM, and the FSI granules were in the form of a calyx campanulate, enclosed by rounded petals, with a cylindrical interior and 10 stamens surrounding the pistil [4]. Figure 2A,B show that the particles of both the VD- and UAVD-treated samples were oval, 6 mm long and 3 mm in diameter. However, the colour of the shells of the samples dried with UAVD differed from that of the control VD samples, in which the VD samples were darker and the shells of the UAVD samples showed a certain yellow-green colour and were full of convex holes. As can be seen in Figure 2C,D, the cross section of UAVD particles is full, while the VD particles are rough. The free water of the VD sample evaporates and boils simultaneously under vacuum conditions. Meanwhile, the evaporation rate is accelerated, and a large pressure gradient is formed between the inner and outer layers of the material and between the surface and the surrounding medium, resulting in a porous structure of the VD dried product. This loose and brittle sponge-like porous structure is easy to crush [6]. In contrast, there were more pores on the surface of the UAVD-treated samples. This phenomenon was due to the formed channels, which allow faster water escape and faster drying time during VD action. The results were consistent with Liu et al. [22].

The SEM microscopic images of the VD and UAVD FSI samples shown are in Figure 2E–H. The pistil structure of the VD samples was disorganized, and the pollen grain structure was fragmented (Figure 2E,G) [23]. The pistil structure of the UAVD sample was intact and the pollen grain structure was more complete, with an average diameter of 70 μm (Figure 2F,H). A large number of pores appeared on the surface of FSI after ultrasonic treatment, which may be due to the destruction of cell wall structure by ultrasound, forming channels that allow water to drain out more easily and prevent the cell structure from being damaged by the intense water dissipation under vacuum conditions [9]. The SEM results validate the SM and MC results, which are consistent with those reported by Shi et al. [24].

### 3.3. Colour Analysis

**Figure 3:**
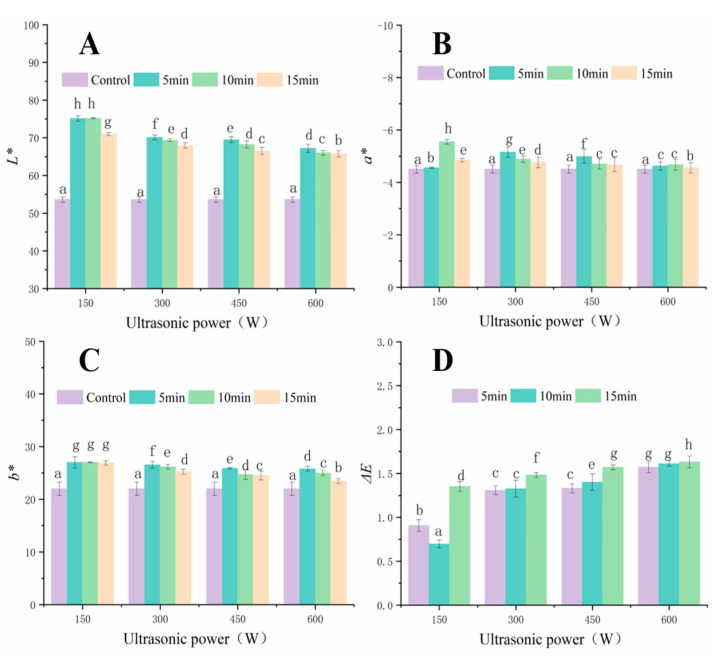
Changes in *L* (**A**), a* (**B**), b* (**C**)*,* and Δ*E* (**D**) for the colour difference in FSI powders obtained by selected differences in ultrasonic power of UAVD. Different letters in the histogram indicate that the extraction time points within UAVD are signif-icantly different (*p* < 0.05).

Colour is a key factor in consumers’ choice of products [22]. As shown in Figure 3, UAVD significantly increased the value of L* (Figure 3A). The L* values gradually decreased with increasing ultrasonic treatment time and power, exhibiting a maximum value at 150 W in 10 min. As shown in Figure 3B, a* values were all less than 0, indicating that the UAVD-treated FSI samples tended to be green. The a* values for samples treated with 150 W for 10 min were lower and greener in colour, and these results may be due to the fact that low-powered ultrasound relaxed the cell walls of the samples, which contributed to the release of chlorophyll, while high-powered ultrasound may have destroyed chlorophyll [25]. Among the colour parameters, the difference in b* was significant (Figure 3C, *p* < 0.05). The b* values were highest for samples treated with 150 W ultrasound for 10 min, indicating that FSI turns yellow during the UAVD process. The highest values of L*, a*, and b* were obtained at 150 W for 10 min. This may be due to the influence of yellow (carotenoids) and green (chlorophyll) colours. In addition, ultrasound causes the escape of flavonoids, which themselves carry a colour that changes the colour of the sample. This result is in agreement with that of de Araujo et al. [26].

Our results showed no significant differences between the Δ*E* parameters of the UAVD and VD samples (Figure 3D). The value of Δ*E* caused by the UAVD treatment increased with increasing ultrasound power. The Δ*E* value of the 150 W ultrasound treatment for 10 min was 0.066 and increased to 1.631 when the ultrasound power and time were increased to 600 W for 15 min. However, the Δ*E* values for all sample groups were less than 2. This result indicated that the ultrasonic treatment had little effect on the colour of the samples [27]. Herein, considering all colour parameters together, there was no significant change in the application of ultrasound in vacuum drying. Thermal effects played a dominant role in the colour change of the dried products. This is in agreement with the findings of Wu et al. [28].

### 3.4. Flavonoid Analysis

**Figure 4:**
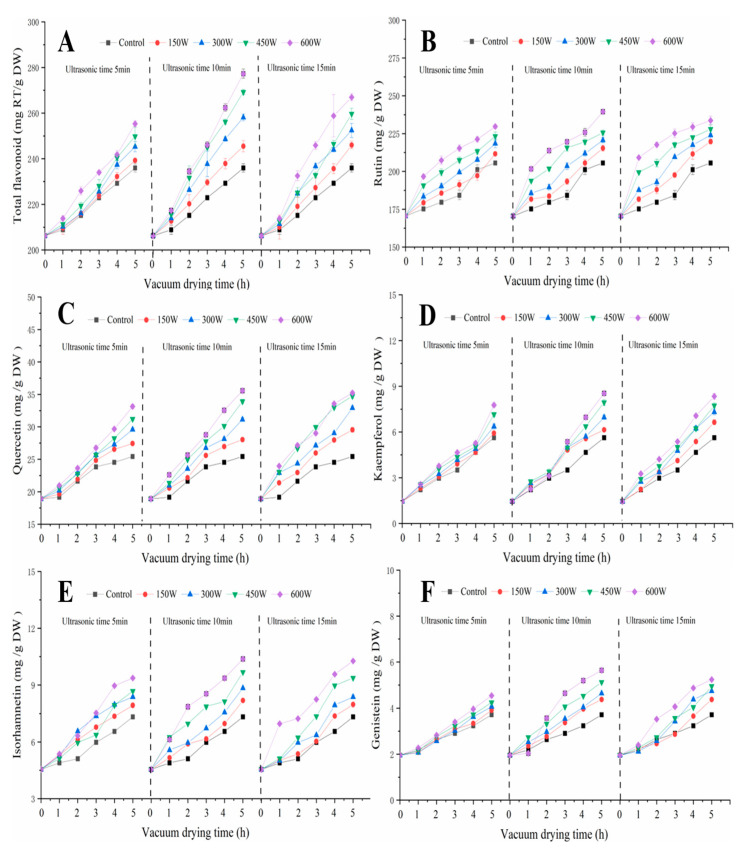
The effects of UAVD on the contents of total flavonoids, rutin, quercetin, kaempferol, isorhamnetin, and genistein (**A**–**F**) in FSI at different time durations.

The positive and negative effects of ultrasound on the TFC were observed [29]. The TFC increased significantly after UAVD treatment (Figure 4A), and the trend of TFC was similar under different UAVD conditions. The highest TFC was found at 600 W for 10 min (increasing from 235.95 mg/g DW to 277.28 mg/g DW) (*p* < 0.05). This may be caused by the proper heat treatment, in which high-power ultrasound breaks covalent bonds and promotes the release of antioxidants, such as flavonoids and phenols [16,30]. However, the TFC at 600 W for 15 min was 266.94 mg/g DW, which may be due to excessive ultrasonic pre-treatment leading to nutrient loss from the food [31].

We also investigated the effect of ultrasound on the contents of flavonoids (Figure 4B–F), and the results showed that the contents increased significantly after UAVD treatment, and the trends in TFC were similar under different UAVD conditions. The highest levels of rutin, quercetin, kaempferol, isorhamnetin, and genistein were found at 600 W for 10 min with 239.46, 35.56, 8.54, 10.37, and 5.64 mg/g DW, respectively. This suggests that ultrasound power and ultrasound time significantly influenced the flavonoid contents. This may be due to ultrasound promoting the release of flavonoids [32,33]. Gong et al. [4] showed similar results in ultrasound-assisted freeze drying. Our results suggest that ultrasound can promote the release of flavonoid bioactive substances from plant materials.

### 3.5. Antioxidant Capacity Analysis

**Figure 5:**
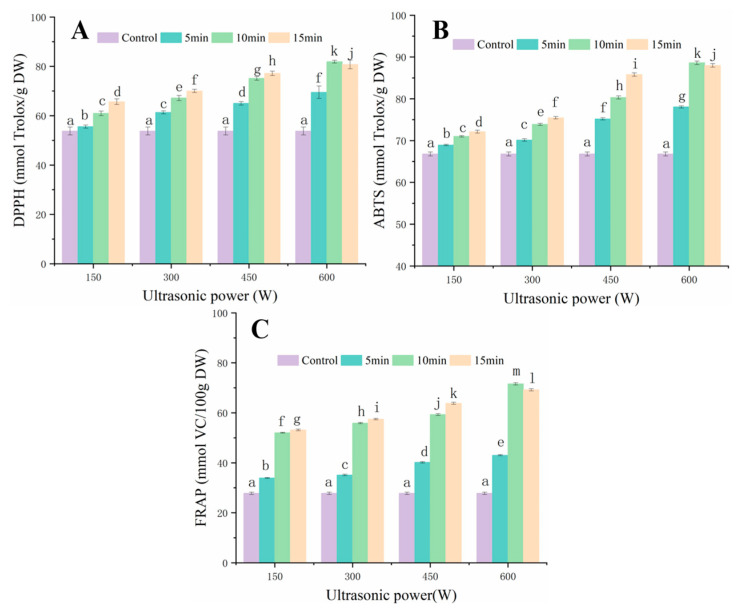
The free radical scavenging rate of FSI. DPPH (**A**); ABTS (**B**); and FRAP (**C**) of the FSI after UAVD. Different letters in the histogram indicate that the extraction time points within UAVD are significantly different (*p* < 0.05).

Studies confirmed that FSI possessed obvious antioxidant bioactivity, as it is rich in rutin, quercetin, and other polyphenols that are beneficial to health [34,35]. To accurately reflect the antioxidant activity of FSI, this study used DPPH, ABTS, FRAP, and three different assays to comprehensively assess the antioxidant capacity of FSI after UAVD treatment [36,37]. The effect of ultrasound on DPPH free radical scavenging ability (RSA) is shown in Figure 5A. The scavenging effect of UAVD on DPPH RSA was significantly higher. The results showed a positive correlation between antioxidant capacity and ultrasound power (*p* < 0.05) [38]. The DPPH RSA was progressively enhanced by a 150 W ultrasound of FSI with increasing pre-treatment time (5, 10, and 15 min) (55.59%, 60.97%, and 65.65%). Kroehnke et al. [39] had similar results with UAVD. Furthermore, the DPPH RSA treated with ultrasound at 300 W, 450 W, and 600 W for a 5 min treatment time gradually increased for DPPH radicals compared to the 150 W treatment (70.03, 77.17, and 80.75%), and the strongest scavenging ability of DPPH radicals (81.86%) was observed in the 600 W, 10 min ultrasound. In contrast, the scavenging ability of FSI samples treated with ultrasound at 600 W for 10 min for DPPH radicals did not change significantly with increasing ultrasound time. The scavenging effect of UAVD on DPPH radicals was compared according to the relationship between ultrasound time and ultrasound power response. The effect of ultrasound power treatment on the scavenging ability of DPPH radicals of different samples was in the order 600 > 450 > 300 > 150 w, and the scavenging ability of DPPH radicals was significantly enhanced. This may be related to the release of flavonoids from FSI after ultrasound treatment [40,41]. Furthermore, there appears to be a synergistic effect between the ultrasound time and the thermal effect, increasing the release of bioactive compounds and ultimately resulting in enhancing the antioxidant capacity [42].

The ABTS RSA of UAVD-treated samples was compared (Figure 5B). With increasing time, the ABTS RSA of the 150 W ultrasound gradually increased. In addition, the RSA of FSI samples treated at 300 W, 450 W, and 600 W for 5 min for ABTS radicals was progressively enhanced compared to the 150 W treatment time. This indicated that the scavenging effect of ABTS radicals was positively correlated with the ultrasound time and power (*p* < 0.05) [43,44]. The treatment with 600 W for 10 min showed the highest ABTS RSA (88.61%). The effect of FSI samples treated with UAVD on ABTS RSA was compared. The effects of ultrasound power treatment on the scavenging ability of ABTS radicals of different samples were in the order of 600 > 450 > 300 > 150 W, with a significant increase in the scavenging ability of ABTS radicals. This may be related to the release of flavonoids from FSI after ultrasound treatment. This is consistent with the findings of Mello et al. [45]. The FRAP RSA gradually increased with time and power after ultrasound (Figure 5C), which is consistent with the results of Dzah et al. [46]. The highest scavenging ability of FRAP (71.61%) was obtained at 600 W for 10 min. The effect of UAVD on FRAP RSA was compared according to the relationship between ultrasound time and ultrasound power response. The order of effect of ultrasound power treatment was 600 > 450 > 300 > 150 W. This may be related to the release of flavonoids from FSI after ultrasound treatment. This result is consistent with Zhang et al. [47]. Tchabo et al. [38] found that ultrasonically treated samples had better antioxidant activity compared to untreated samples. Therefore, these results suggest that moderate UAVD treatment has a positive impact on the ability of FSI to resist DPPH, ABTS, and FRAP.

### 3.6. Effect of UAVD on α-Glucosidase and α-Amylase Inhibition Capacity of FSI

**Figure 6:**
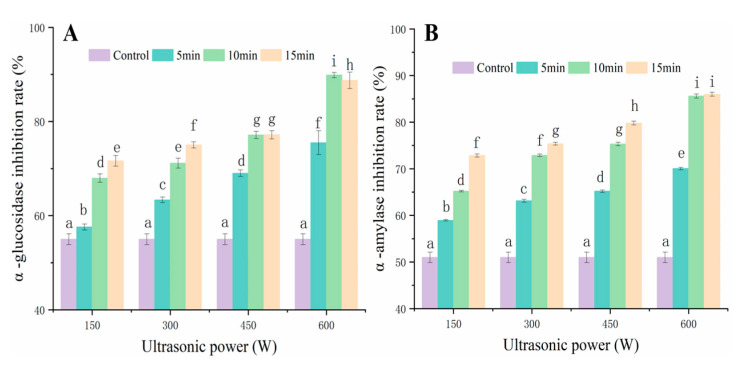
Effect of UAVD treatment on α-glucosidase (**A**) and α-amylase (**B**) inhibition capacity of FSI. Different letters in the histogram indicate that the extraction time points within UAVD are significantly different (*p* < 0.05).

Flavonoids in FSI can delay glucose uptake and reduce blood glucose levels by inhibiting α-glucosidase and α-amylase activities [48,49,50,51]. As shown in Figure 6A, the α-glucosidase inhibition rate was 55% after VD treatment. The α-glucosidase inhibition rate was gradually increased to 75% with the increase in ultrasound power to 600 W for 5 min, which may be due to the elevated levels of bioactive compounds such as rutin and quercetin after UAVD. Moreover, the easy interaction of quercetin with amino acid residues in the important catalytic sites of α-glucosidase and α-amylase was shown, in addition to the inhibition activity of α-glucosidase. Zhu et al. [52] reported that in Ascophyllum nodosum drying, ultrasound-assisted treatment could promote the release of TFC during drying compared to convective drying without ultrasound. However, at an ultrasound power of 600 W, the inhibition rates of α-glucosidase were 75, 89, and 88% with increasing ultrasound time to 5, 10, and 15 min, respectively. It could be that a short time at the same ultrasound power could promote the release of flavonoids during UAVD and that a too-long ultrasound time had no effect. This is consistent with the results of Xie et al. [53]. The inhibition rate of α-amylase during UAVD was similar to that of α-glucosidase (Figure 6B). The inhibition rate of α-amylase gradually increased with the increase in power, showing that the inhibition rates of 150 W, 300 W, 450 W, and 600 W were 58%, 63%, 65%, and 70%, respectively, at 5 min of ultrasound time. At 600 W, the inhibition rates of α-glucosidase were 70, 85, and 85% with increasing ultrasound time from 5, 10, and 15 min, respectively. This result is in agreement with the results of Ismail et al. [18]. The results indicated that the inhibitions of α-glucosidase and α-amylase by ultrasound during UAVD were significantly increased.

## 4. Conclusions

In the present study, UAVD significantly reduced the drying time of FSI (40%) compared to VD techniques. Micrographs showed that UAVD created more porous structures and accelerated the migration of water from the inside to the outside of the FSI tissue. The flavonoid content of UAVD products was superior to other conditions at 600 W for 10 min. In addition, the low ΔE, antioxidant activity, and α-glucosidase and α-amylase inhibition capacities were the highest characteristic traits under this condition. This may be the channel formed by the ultrasonic cavitation effect, which can promote the release of flavonoids. The results show that UAVD can significantly shorten the drying time, promote the release of bioactive substances in FSI, enhance the antioxidant, α-glucosidase, and α-amylase inhibition capacities of FSI, and is a promising drying method.

## Data Availability

The data presented in this study are available on request from the corresponding author.

## References

[1] Gong Y., Fan L., Wang L., Li J. (2021). *Flos Sophorae Immaturus*: Phytochemistry, bioactivities, and its potential applications. Food Rev. Int..

[2] Li J., Gong Y., Li J., Fan L. (2022). In vitro xanthine oxidase inhibitory properties of *Flos Sophorae Immaturus* and potential mechanisms. Food Biosci..

[3] Gan Z., Chen Q., Fu Y., Chen G. (2012). Determination of bioactive constituents in *Flos Sophorae Immaturus* and Cortex Fraxini by capillary electrophoresis in combination with far infrared-assisted solvent extraction. Food Chem..

[4] Gong Y., Li J., Li J., Fan L., Wang L. (2022). Effect of ultrasound-assisted freeze-dried on microstructure, bioactive substances, and antioxidant activity of *Flos Sophorae Immaturus*. Food Biosci..

[5] He X., Bai Y., Zhao Z., Wang X., Fang J., Huang L., Zeng M., Zhang Q., Zhang Y., Zheng X. (2016). Local and traditional uses, phytochemistry, and pharmacology of *Sophora japonica L.*: A review. J. Ethnopharmacol..

[6] Yu Q., Li J., Fan L. (2019). Effect of drying methods on the microstructure, bioactivity substances, and antityrosinase activity of asparagus stems. J. Agric. Food Chem..

[7] Li Y., Wang X., Wu Z., Wan N., Yang M. (2020). Dehydration of hawthorn fruit juices using ultrasound-assisted vacuum drying. Ultrason. Sonochem..

[8] Colucci D., Fissore D., Rossello C., Carcel J.A. (2018). On the effect of ultrasound-assisted atmospheric freeze-drying on the antioxidant properties of eggplant. Food Res. Int..

[9] Baeghbali V., Ngadi M., Niakousari M. (2020). Effects of ultrasound and infrared assisted conductive hydro-drying, freeze-drying and oven drying on physicochemical properties of okra slices. Innov. Food Sci. Emerg. Technol..

[10] Pei Y., Li Z., Xu W., Song C., Li J., Song F. (2021). Effects of ultrasound pretreatment followed by far-infrared drying on physicochemical properties, antioxidant activity and aroma compounds of saffron (*Crocus sativus L.*). Food Biosci..

[11] Ricce C., Rojas M.L., Miano A.C., Siche R., Augusto P.E.D. (2016). Ultrasound pre-treatment enhances the carrot drying and rehydration. Food Res. Int..

[12] Wang G., Cui Q., Yin L.-J., Li Y., Gao M.-Z., Meng Y., Li J., Zhang S.-D., Wang W. (2020). Negative pressure cavitation based ultrasound-assisted extraction of main flavonoids from *Flos Sophorae Immaturus* and evaluation of its extraction kinetics. Sep. Purif. Technol..

[13] Zhang Y., Abatzoglou N. (2020). Review: Fundamentals, applications and potentials of ultrasound-assisted drying. Chem. Eng. Res. Des..

[14] Liu Y., Tian J., Hu B., Yu P., Fan L. (2021). Relationship between crust characteristics and oil uptake of potato strips with hot-air pre-drying during frying process. Food Chem..

[15] Liu Y., Tian J., Zhang T., Fan L. (2021). Effects of frying temperature and pore profile on the oil absorption behavior of fried potato chips. Food Chem..

[16] Yao L., Fan L., Duan Z. (2020). Effect of different pretreatments followed by hot-air and far-infrared drying on the bioactive compounds, physicochemical property and microstructure of mango slices. Food Chem..

[17] Xiao Y., Yang C., Xu H., Zhang J., Zhang L. (2021). Study on the change of flavonoid glycosides to aglycones during the process of steamed bread containing tartary buckwheat flour and antioxidant, α-glucosidase inhibitory activities evaluation in vitro. LWT-Food Sci. Technol..

[18] Ismail B.B., Liu D., Pu Y., He Q., Guo M. (2021). High-intensity ultrasound processing of baobab fruit pulp: Effect on quality, bioactive compounds, and inhibitory potential on the activity of alpha-amylase and alpha-glucosidase. Food Chem..

[19] Guo Y., Wu B., Guo X., Ding F., Pan Z., Ma H. (2020). Effects of power ultrasound enhancement on infrared drying of carrot slices: Moisture migration and quality characterizations. LWT-Food Sci. Technol..

[20] Huang D., Men K., Li D., Wen T., Gong Z., Sunden B., Wu Z. (2020). Application of ultrasound technology in the drying of food products. Ultrason. Sonochem..

[21] Yao Y. (2016). Enhancement of mass transfer by ultrasound: Application to adsorbent regeneration and food drying/dehydration. Ultrason. Sonochem..

[22] Liu Y.-Y., Wang Y., Lv W.-Q., Li D., Wang L.-J. (2021). Freeze-thaw and ultrasound pretreatment before microwave combined drying affects drying kinetics, cell structure and quality parameters of *Platycodon grandiflorum*. Ind. Crops Prod..

[23] Smyth A.P., Seales B., Bradley P.M. (2015). A pollen profile by scanning electron microscopy bracketing the mid-holocene tsuga canadensis decline at Poutwater Pond Bog, Holden, Massachusetts. Grana.

[24] Shi X., Yang Y., Li Z., Wang X., Liu Y. (2020). Moisture transfer and microstructure change of banana slices during contact ultrasound strengthened far-infrared radiation drying. Innov. Food Sci. Emerg. Technol..

[25] Li L., Yu Y., Xu Y., Wu J., Yu Y., Peng J., An K., Zou B., Yang W. (2021). Effect of ultrasound-assisted osmotic dehydration pretreatment on the drying characteristics and quality properties of Sanhua plum (*Prunus salicina L.*). LWT-Food Sci. Technol..

[26] de Araujo F.F., de Paulo Farias D., Neri-Numa I.A., Dias-Audibert F.L., Delafiori J., de Souza F.G., Catharino R.R., do Sacramento C.K., Pastore G.M. (2021). Influence of high-intensity ultrasound on color, chemical composition and antioxidant properties of araca-boi pulp. Food Chem..

[27] Santos K.C., Guedes J.S., Rojas M.L., Carvalho G.R., Augusto P.E.D. (2021). Enhancing carrot convective drying by combining ethanol and ultrasound as pre-treatments: Effect on product structure, quality, energy consumption, drying and rehydration kinetics. Ultrason. Sonochem..

[28] Wu B., Guo X., Guo Y., Ma H., Zhou C. (2021). Enhancing jackfruit infrared drying by combining ultrasound treatments: Effect on drying characteristics, quality properties and microstructure. Food Chem..

[29] Qi Y., Sun A., Liu R., Meng Z., Xie H. (2007). Isolation and purification of flavonoid and isoflavonoid compounds from the pericarp of *Sophora japonica L.* by adsorption chromatography on 12% cross-linked agarose gel media. J. Chromatogr. A.

[30] Zhang L., Qiao Y., Liao L., Shi D., An K., Jun W., Liu S. (2021). Effects of ultrasound and ultra-high pressure pretreatments on volatile and taste compounds of vacuum-freeze dried strawberry slice. Lwt.

[31] Stojanovic J., Silva J.L. (2007). Influence of osmotic concentration, continuous high frequency ultrasound and dehydration on antioxidants, colour and chemical properties of rabbiteye blueberries. Food Chem..

[32] Gani A., Baba W.N., Ahmad M., Shah U., Khan A.A., Wani I.A., Masoodi F.A., Gani A. (2016). Effect of ultrasound treatment on physico-chemical, nutraceutical and microbial quality of strawberry. LWT-Food Sci. Technol..

[33] Kek S.P., Chin N.L., Yusof Y.A. (2013). Direct and indirect power ultrasound assisted pre-osmotic treatments in convective drying of guava slices. Food Bioprod. Process..

[34] Li Y., Fan L. (2020). Comparative studies on the stabilization of *Flos Sophorae Immaturus* beverages by various hydrocolloids. LWT-Food Sci. Technol..

[35] Liao J., Qu B., Liu D., Zheng N. (2015). New method to enhance the extraction yield of rutin from *Sophora japonica* using a novel ultrasonic extraction system by determining optimum ultrasonic frequency. Ultrason. Sonochem..

[36] Brand-Williams W., Cuvelier M.E., Berset C. (1995). Use of a free radical method to evaluate antioxidant activity. LWT-Food Sci. Technol..

[37] Qi Y., Yu F., Wang X., Wan N., Yang M., Wu Z., Li Y. (2021). Drying of wolfberry fruit juice using low-intensity pulsed ultrasound. LWT-Food Sci. Technol..

[38] Tchabo W., Ma Y., Kwaw E., Zhang H., Li X., Afoakwah N.A. (2017). Effects of ultrasound, high pressure, and manoultrasound processes on phenolic profile and antioxidant properties of a sulfur dioxide-free mulberry *(Morus nigra*) wine. Food Bioprocess Technol..

[39] Kroehnke J., Szadzinska J., Radziejewska-Kubzdela E., Bieganska-Marecik R., Musielak G., Mierzwa D. (2021). Osmotic dehydration and convective drying of kiwifruit (*Actinidia deliciosa*)—The influence of ultrasound on process kinetics and product quality. Ultrason. Sonochem..

[40] Santos N.C., Almeida R.L.J., da Silva G.M., Monteiro S.S., André A.M.M.C.N. (2020). Effect of ultrasound pre-treatment on the kinetics and thermodynamic properties of guava slices drying process. Innov. Food Sci. Emerg. Technol..

[41] Li X., Zhang L., Peng Z., Zhao Y., Wu K., Zhou N., Yan Y., Ramaswamy H.S., Sun J., Bai W. (2020). The impact of ultrasonic treatment on blueberry wine anthocyanin color and its in-vitro anti-oxidant capacity. Food Chem..

[42] Tao Y., Li D., Siong Chai W., Show P.L., Yang X., Manickam S., Xie G., Han Y. (2021). Comparison between airborne ultrasound and contact ultrasound to intensify air drying of blackberry: Heat and mass transfer simulation, energy consumption and quality evaluation. Ultrason. Sonochem..

[43] Xu X., Zhang L., Feng Y., Zhou C., Yagoub A.E.A., Wahia H., Ma H., Zhang J., Sun Y. (2021). Ultrasound freeze-thawing style pretreatment to improve the efficiency of the vacuum freeze-drying of okra (*Abelmoschus esculentus (L.) Moench*) and the quality characteristics of the dried product. Ultrason. Sonochem..

[44] Mahindrakar K.V., Rathod V.K. (2022). Ultrasound-assisted intensified aqueous extraction of phenolics from waste Syzygium cumini leaves: Kinetic studies and evaluation of antioxidant, antidiabetic and anticancer potential. Food Biosci..

[45] Mello R.E., Fontana A., Mulet A., Corrêa J.L.G., Cárcel J.A. (2021). PEF as pretreatment to ultrasound-assisted convective drying: Influence on quality parameters of orange peel. Innov. Food Sci. Emerg. Technol..

[46] Dzah C.S., Duan Y., Zhang H., Boateng N.A.S., Ma H. (2020). Ultrasound-induced lipid peroxidation: Effects on phenol content and extraction kinetics and antioxidant activity of Tartary buckwheat (*Fagopyrum tataricum*) water extract. Food Biosci..

[47] Zhang C., Liu D., Gao H. (2018). Kinetics, physicochemical properties, and antioxidant activities of Angelica keiskei processed under four drying conditions. LWT-Food Sci. Technol..

[48] Oboh G., Ademosun A.O., Ayeni P.O., Omojokun O.S., Bello F. (2014). Comparative effect of quercetin and rutin on α-amylase, α-glucosidase, and some pro-oxidant-induced lipid peroxidation in rat pancreas. Comp. Clin. Pathol..

[49] Ji Y., Liu D., Jin Y., Zhao J., Zhao J., Li H., Li L., Zhang H., Wang H. (2021). In vitro and in vivo inhibitory effect of anthocyanin-rich bilberry extract on α-glucosidase and α-amylase. LWT-Food Sci. Technol..

[50] Li M., Bao X., Zhang X., Ren H., Cai S., Hu X., Yi J. (2022). Exploring the phytochemicals and inhibitory effects against α-glucosidase and dipeptidyl peptidase-IV in chinese pickled chili pepper: Insights into mechanisms by molecular docking analysis. LWT-Food Sci. Technol..

[51] Liu S., Yu J., Guo S., Fang H., Chang X. (2020). Inhibition of pancreatic α-amylase by Lonicera caerulea berry polyphenols in vitro and their potential as hyperglycemic agents. LWT-Food Sci. Technol..

[52] Zhu X., Zhang Z., Hinds L.M., Sun D.W., Tiwari B.K. (2021). Applications of ultrasound to enhance fluidized bed drying of Ascophyllum Nodosum: Drying kinetics and product quality assessment. Ultrason. Sonochem..

[53] Xie Z., Sun Y., Lam S., Zhao M., Liang Z., Yu X., Yang D., Xu X. (2014). Extraction and isolation of flavonoid glycosides from *Flos Sophorae Immaturus* using ultrasonic-assisted extraction followed by high-speed countercurrent chromatography. J. Sep. Sci..

